# Urban–Rural Differences in Preferences for Environmentally Friendly Farming from the Perspectives of Oriental White Stork Conservation

**DOI:** 10.3390/ani16020318

**Published:** 2026-01-20

**Authors:** Liyao Zhang, Zhen Miao, Yinglin Wang, Xingchun Li, Xuehong Zhou, Yujuan Gao

**Affiliations:** 1 College of Economics and Management, Northeast Forestry University, Harbin 150040, China; Xybzly@126.com; 2 College of Wildlife and Protected Area, Northeast Forestry University, Harbin 150040, China; miaozhen43566@163.com (Z.M.); 17616079117@163.com (Y.W.); Lixingchun0612@126.com (X.L.)

**Keywords:** wildlife conservation, the Oriental White Stork, wildlife-friendly farming, differences in preference, ecological products

## Abstract

Expanded and intensified agriculture is a major driver of habitat loss for endangered species such as the Oriental White Stork (*Ciconia boyciana*), making wildlife-friendly farming an increasingly important approach for reconciling biodiversity conservation with agricultural development. Building on a 2018 feasibility study in the Sanjiang Plain, this research employs a choice experiment to examine how preferences for Oriental White Stork-friendly farming have evolved among urban consumers and residents of stork habitats under expanding green consumption and increasing experience with environmentally friendly farming. The results reveal pronounced preference heterogeneity and persistent cognitive separation between wildlife conservation and agricultural production, particularly among urban consumers, despite a stable group being willing to pay a premium for stork-friendly products. Rural residents’ decisions remain largely economically driven, though younger farmers with prior experience in environmentally friendly practices show more positive attitudes. Significant urban–rural differences suggest policy complementarities, whereby price-oriented incentives may encourage price-sensitive farmers to adopt green agriculture, while intrinsically motivated farmers require support through an Oriental White Stork-oriented value chain. Overall, the findings demonstrate that Wildlife-Friendly Farming cannot be effectively promoted through a one-size-fits-all approach; instead, stratified, group-specific policy and market mechanisms are essential for aligning producer incentives with consumer demand and supporting the long-term viability of biodiversity-friendly agricultural systems.

## Simple Summary

The expansion of rice farming in China’s Sanjiang Plain threatens the survival of the endangered Oriental White Stork. “Wildlife-friendly farming,” which allows birds to forage in rice paddies, offers a promising solution, but its success depends on the joint support of farmers and consumers. However, existing research has largely focused on single groups, lacking direct comparisons or long-term perspectives on their differing preferences. This study investigated whether local farmers are willing to adopt bird-friendly practices and if urban consumers are interested in purchasing the resulting rice. We discovered a surprising conflict: while many urban residents are willing to pay extra for green or organic food, they paradoxically reject the presence of wild birds in fields, likely due to food safety or hygiene concerns. Meanwhile, farmers are primarily motivated by higher selling prices. Our findings suggest that connecting farmers who need higher incomes with consumers willing to pay for premium products can bridge this gap. This study highlights how correcting public misconceptions can facilitate the prosperous coexistence of endangered wildlife and sustainable agriculture.

## 1. Introduction

The rapid expansion of cropland and the intensification of agricultural production are prominent features of global land-use change [1], acting as primary drivers of wildlife habitat loss and biodiversity decline [2,3]. Driven by population growth and rising food demand [4], the tension between agricultural production goals and wildlife conservation has continued to escalate across many regions [5]. Global-scale comprehensive analyses indicate that agricultural activities have exerted significant pressure on the survival of numerous threatened species; among the 8688 species evaluated on the IUCN Red List, approximately 62% (*n* = 5407) are currently threatened by agricultural practices [6]. However, agricultural development and wildlife conservation are not inherently antithetical. Increasing evidence suggests that under specific landscape and management scenarios, farmland can provide supplementary habitats and resources for certain wildlife species, with its ecological functions complementing those of natural habitats to a certain extent [7,8]. Therefore, the critical challenge lies in identifying scientific strategies that can safeguard agricultural output while simultaneously mitigating adverse impacts on wildlife and enhancing the supportive functions of these habitats.

Wildlife-Friendly Farming (WFF) offers a critical pathway for mitigating the aforementioned conflicts. As an emerging agricultural paradigm and a subset of environmentally friendly agriculture, WFF emphasizes enhancing the biodiversity carrying capacity of farmland landscapes by integrating specific management interventions while maintaining or optimizing agricultural yields [9]. In contrast to intensive agricultural models that advocate for the strict segregation of production and conservation (“land-sparing”), WFF embodies the core concept of “land-sharing,” advocating for the integration of ecological conservation processes directly within agricultural production [10]. To date, WFF has been implemented across various wildlife taxa, including birds, mammals, reptiles, amphibians, and fish [11,12,13,14]. It has demonstrated significant potential in advancing wildlife conservation, restoring agricultural landscapes, and driving agricultural economic development [15].

Wildlife-friendly farming is essentially a complex social-ecological system (SES) [16]. Its successful implementation depends not only on effective agro-ecological techniques but also, and more critically, on stakeholder value judgments and social acceptability [17]. Within this context, behavioral economic theories provide a foundation for understanding such complex social decision-making processes. The Theory of Planned Behavior (TPB) offers a widely utilized framework for explaining how individual attitudes toward environment-friendly practices shape preferences and choices, thereby elucidating how these attitudes influence the feasibility of sustainable agricultural practices [18,19]. Furthermore, Environmental Value Theory posits that individuals hold relatively stable value orientations—specifically biospheric, altruistic, or egoistic values [20]. These orientations influence the assessment and prioritization of environmental issues, forming a crucial cognitive link between agricultural sustainability and broader conservation objectives.

Building on these theoretical frameworks, numerous studies have employed preference elicitation and perception analysis methods to quantify how various stakeholder groups perceive environmental benefits, navigate trade-offs, and assume responsibility [18,21], thereby identifying critical entry points for conservation policy [21]. Recent empirical research confirms that the urban–rural divide plays a particularly significant role in the formation of environmental perceptions. Disparities in environmental knowledge, income levels, degree of dependence on natural resources, and exposure to environmental risks have shaped distinct perception pathways within urban and rural contexts [22]. Rural residents—particularly those directly engaged in agriculture—often evaluate environmental measures through the lens of lived experience, place-based attributes, and cost–benefit considerations [23]. In contrast, urban residents are more likely to participate in wildlife conservation driven by ethics, cultural beliefs, or ecological values [24]. These divergent perceptions may directly lead to variations in the social acceptability of conservation measures for endangered species among different stakeholder groups, and further determine the degree of support and Willingness to Pay (WTP) for wildlife-friendly farming practices across these populations. Consequently, quantifying the differences in attitudes and preferences between groups and identifying their underlying psychological drivers is a vital step toward establishing equitable and effective WFF mechanisms.

The Oriental White Stork (*Ciconia boyciana*), belonging to the order Ciconiiformes and the family Ciconiidae, is listed as Endangered (EN) by the International Union for Conservation of Nature (IUCN). Valued for its ecological importance and high sensitivity to environmental change, it is widely recognized as an indicator species [25]. The distribution of the Oriental Stork is restricted to Asia, with the Sanjiang Plain in Northeast China serving as its primary breeding ground and habitat. Historical research indicates that in Northeast Asian breeding regions, the Oriental Stork prefers nesting in marshy wetlands and foraging in marshes or mudflats with extensive shallow water. However, as natural wetlands undergo degradation and fragmentation, artificial wetlands—such as paddy fields and aquaculture ponds—have increasingly become critical alternative foraging habitats. This shift has directly led to intensified conflicts between the Oriental Stork and farmers in areas surrounding its natural habitats. Because the storks frequently forage in rice paddies and fish ponds, local farmers bear the costs associated with conservation, while the species itself faces heightened risks from agricultural pollution, human disturbance, and other environmental stressors [3]. Since traditional ecological compensation mechanisms often rely heavily on single-source, unsustainable government fiscal resources, promoting an “Oriental Stork-Friendly Farming” model, which balances conservation with agricultural development, has become an essential requirement for advancing the protection of this endangered species.

In recent years, China’s domestic market for green and organic food has expanded rapidly, becoming the fourth-largest market globally [26]. Consumer priorities have shifted from meeting basic needs to emphasizing product quality [27], and studies consistently report rising expectations for food quality and safety standards [28,29,30]. As one of the country’s most vital grain-producing regions, the Sanjiang Plain has intensified its support for the green and organic sectors in response to steady market growth. By 2025, the number of green-food-certified enterprises in the region reached 1158, and the proportion of green and organic certified cropland rose to 42.2% (totaling 28.45 million mu) [31]. Leveraging the unique natural resource advantages of being the “Home of the Oriental Stork,” the region has developed specialized rice brands by promoting organic practices such as rice–fish and rice–crab co-culture systems. These initiatives have supported agricultural upgrading and established a solid foundation for the development of Oriental Stork-friendly farming [3]. Given the momentum of green agriculture and the urgency of stork conservation, our research team conducted a preliminary study in 2018 regarding the tensions between conservation and cultivation. The results demonstrated that despite persistent issues with consumer trust and technical deficiencies, integrated models such as rice–fish and rice–loach systems remain viable and are capable of garnering consumer confidence [3].

However, despite the foundation provided by China’s booming green consumption market for Oriental Stork-friendly farming, stakeholder attitudes and preferences are inherently dynamic. Most previous studies have focused on feasibility analyses within existing contexts, often overlooking how the psychological attitudes and Willingness to Pay (WTP) of urban consumers and rural growers may shift in response to policy changes, technological advancements, and market maturation. As a follow-up investigation, this study builds upon existing wildlife-friendly farming initiatives and prior research to explore how preferences for Oriental Stork-friendly agricultural practices have evolved among both urban consumers and residents within stork habitats. This analysis is situated against a backdrop of expanding green consumption and increasing local experience with environmentally friendly cultivation. By examining preference heterogeneity across both temporal and spatial dimensions, this study assesses the feasibility and challenges of implementing Oriental Stork-friendly farming in the region, offering critical insights to inform evidence-based policy formulation.

## 2. Materials and Methods

### 2.1. Survey Sites and Questionnaires Distribution

This study was conducted in the Sanjiang Plain, Heilongjiang Province, a region crucial for China’s food security and the economic development of Heilongjiang. In 2020, the Plain encompassed 108,900 km^2^ of cultivated land, producing 15 million tons of grain—well above the national average. As Asia’s largest freshwater wetland, the Sanjiang Plain represents a typical inland freshwater marsh and lies along both the Central Asian and West Pacific bird migration flyways [32]. Since the 1990s, nearly one-quarter of the global Oriental White Stork population has bred in the Sanjiang Plain. During spring and autumn migrations, large numbers of the Oriental White Stork forage on fish cultivated in rice paddies, posing significant challenges for local rice farmers [3]. This study designated the Honghe National Nature Reserve (HNNR), the most representative site in the Sanjiang Plain, as the rural study area. HNNR boasts the highest breeding population density of the Oriental Stork globally and is honored as the “Home of the Oriental Stork in China.” Furthermore, situated in the heart of the Sanjiang Plain, the reserve serves as a vital commodity grain production base; thus, the selection of this study site is characterized by significant representativeness.

Since consumers’ willingness to buy also affects the feasibility of Oriental White Stork-friendly farming, this study surveyed potential consumers of such products. Considering the characteristics of China Northeast rice market and consumer interest in organic and green rice, and drawing on the 2019 China Rice Industry Report and the 2021 Tmall Rice Consumption White Paper, East and South China emerged as the primary consumption regions, while online rice consumption has rapidly expanded in Northeast and North China. Analysis of Baidu Index search trends for keywords such as ‘organic rice’ and ‘green rice’ further indicated that Beijing, Harbin, and Hangzhou were high-frequency rice consumption areas with strong interest in environmentally friendly rice. These three cities were therefore selected as the survey sites for this study.

**Figure 1:**
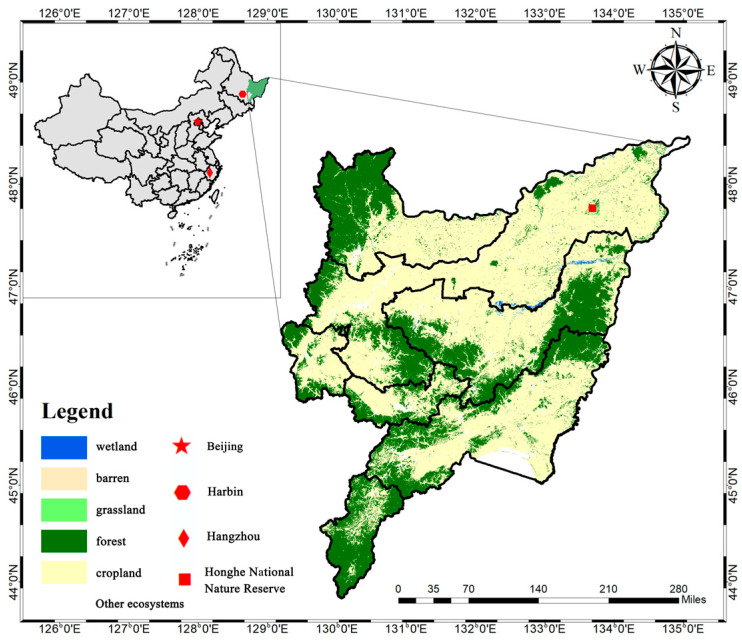
Overview of the study area.

From June to August 2024, this study employed simple random sampling to conduct field surveys and online investigations targeting rural residents in the Honghe National Nature Reserve (HNNR) and potential urban consumers. First, field investigations were conducted within the HNNR between June and July. Following extensive consultation with local wildlife management authorities, sub-regions were identified in areas where rice farmers experienced severe crop losses. Within these zones, rural respondents were selected using simple random sampling. To bolster trust between participants and researchers, local farmers were recruited as field guides. Data collection involved face-to-face, one-on-one interviews, with enumerators recording responses directly into the questionnaires to ensure data quality. Second, an online survey targeting urban consumers was administered via the WJX (WJX.cn) platform from June to August 2024, also utilizing simple random sampling. To safeguard the reliability of the online data, attention-check questions (trap questions) were embedded, and a minimum completion time of 300 s was enforced. Questionnaires that failed to meet either of these criteria were excluded from the analysis as invalid. Based on the 2020 China Land Cover Dataset (CLCD) published by Wuhan University (with a spatial resolution of 30 m and an accuracy of 80%) [33], the natural ecosystem types were identified and the map of the study area was created (Figure 1). The procedures were conducted in ArcGIS 10.8.1.

### 2.2. Research Design

Based on prior research, this study developed tailored questionnaires for rural and urban respondents while maintaining a comparable structure. Section 1 provided an introduction, outlining the conservation status of the Oriental White Stork conservation, including pollution-free, green, and organic methods. Section 2 collected respondents’ socio-economic information, such as gender, age, education level, and annual household income. Section 3 focused on respondent-specific variables: rural participants reported on land-use characteristics (e.g., rice acreage, cultivation costs and income, prior adoption of green or organic practices), as well as their willingness to engage in Oriental White Stork-friendly farming and their market expectations; urban participants were asked about their awareness of ecological agricultural products, consumption behavior (frequency, channels, motivations), and knowledge of and willingness to buy Oriental White Stork-friendly products. Section 4 constitutes the core of this research, presenting the Discrete Choice Experiment (DCE) designed to investigate the preferences of urban and rural respondents regarding Oriental Stork-friendly farming models. Rooted in neoclassical economics, the DCE framework integrates Lancaster’s Theory of Value (Characteristics Theory) with Random Utility Theory (RUT) [34]. The fundamental premise of the choice experiment is that the utility an individual derives from a product does not stem directly from the product as a whole, but rather from its constituent attributes and their specific levels. In a DCE, respondents are required to choose between different “goods” (alternatives) composed of varying attributes and levels. This approach provides a rigorous, direct, and realistic simulation of actual market purchasing decisions, offering superior methodological advantages over traditional stated-preference methods [35]. The attributes and levels selected for this study were primarily informed by preliminary research and an extensive literature review; the specific rationales and definitions for these selections are detailed in Table 1.

Using the selected attributes and levels for the choice experiment, an orthogonal design was generated in SPSS 26.0 (Table 1). To avoid an excessive number of combinations in a full factorial design, clearly implausible or meaningless options were removed, resulting in 12 choice cards. Each card presented two planting options (Option A and Option B) or allowed respondents to indicate a preference for neither. To minimize respondent burden and cognitive load, both rural and urban participants were asked to complete six cards randomly drawn from the full set.

### 2.3. Data Analysis

**Figure 2:**
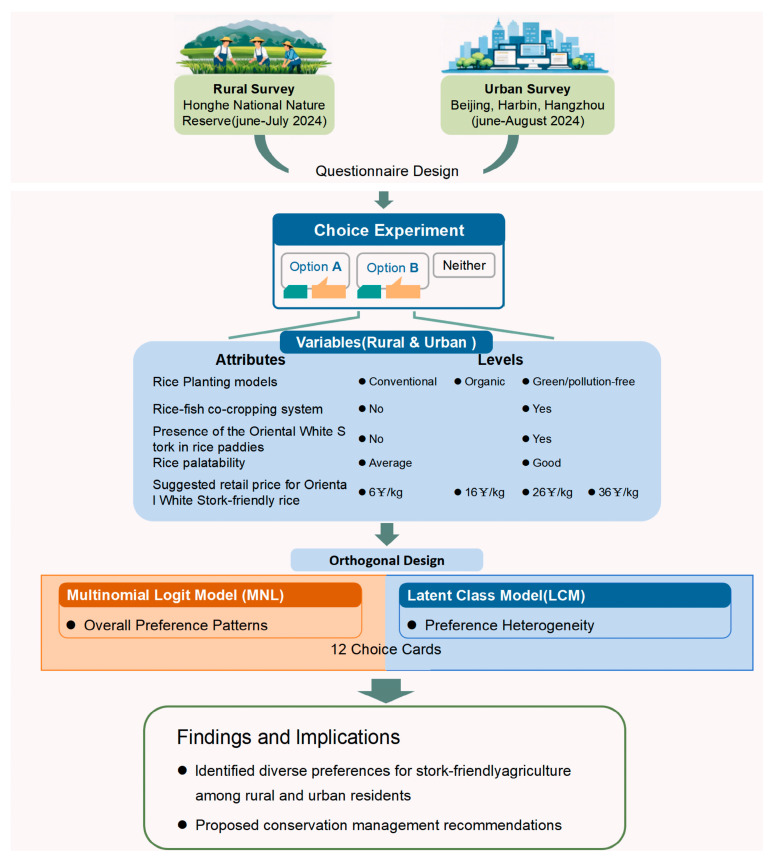
Methodological framework of the study.

Descriptive analyses were conducted using SPSS 26.0 to examine respondents’ socio-economic characteristics, rural respondents’ land-use features and willingness to adopt Oriental White Stork-friendly farming along with market expectations, and urban respondents’ awareness of and behavior toward ecological agricultural products and Oriental White Stork-friendly farming. Chi-square tests assessed whether rural respondents’ market expectations were influenced by factors such as age, education level, gender, household income, and prior farming experience. Preferences for Oriental White Stork-friendly rice planting models among rural and urban respondents were analyzed using Multinomial Logit (MNL) models and Latent Class Models (LCM) in NLOGIT 4.0 to capture both overall patterns and heterogeneity. The latent class model (LCM) is a semi-parametric model that does not require any specific assumptions about the distribution of parameters across individuals. It can use the parameters identified for each segment to explain the sources of heterogeneity. Different socio-demographic characteristics are associated with distinct consumption preferences, which has important implications for policy and management. In contrast, the multinomial logit (MNL) model, which forms the basis of choice experiments, provides a clear overview of the overall preferences of the entire surveyed population. The optimal number of classes in the LCM was determined by balancing the Akaike Information Criterion (AIC) and Bayesian Information Criterion (BIC). The overall methodological framework and the logical flow of data analysis are illustrated in Figure 2.

## 3. Results

By combining face-to-face interviews in rural areas with online questionnaires in urban areas, the study obtained 220 valid rural responses (220 collected; 100% response rate) and 802 valid urban responses (908 collected; 88.33% response rate), yielding 1022 valid responses in total.

### 3.1. Willingness of Residents in the Oriental White Stork Habitat to Accept Oriental White Stork-Friendly Farming and Influencing Factors

**Figure 3:**
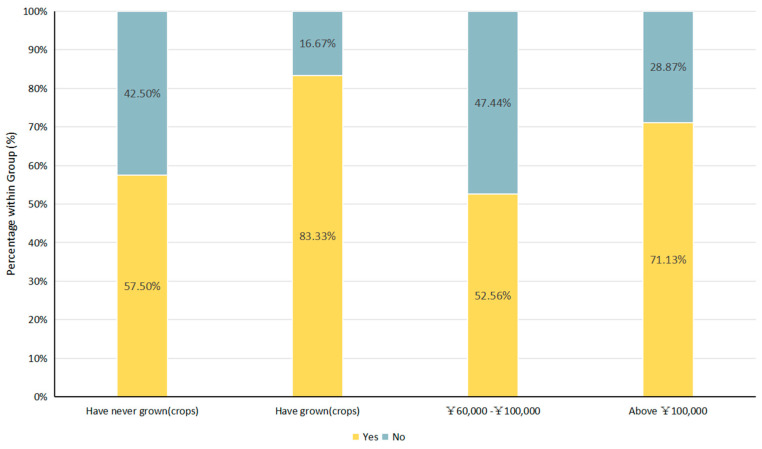
Willingness to adopt Oriental White Stork-friendly agriculture among residents in stork habitats. Note: The Y-axis represents the percentage of respondents within each socioeconomic group. “Yes” indicates an optimistic outlook toward stork-friendly rice production, while “No” indicates a lack of optimism.

A substantial minority of rural respondents reported prior experience with environmentally friendly practices. Specifically, 27.27% had previously planted organic rice, green/pollution-free rice, or adopted a loach–rice co-cropping system. Among these, roughly half (49.09%) reported moderate benefits, while 34.55% perceived positive outcomes. Figure 3 summarizes how such prior experience and household income relate to farmers’ acceptance of Oriental White Stork-friendly farming. Prior experience with green or organic farming significantly influenced willingness to adopt Oriental White Stork-friendly farming. Respondents with experience in planting organic rice were more likely to maintain optimistic expectations about Oriental White Stork-friendly rice (64.55%), with acceptance strongly shaped by past experience and economic status. Specifically, 83.33% of respondents with experience in green, organic, or loach-rice farming expressed positive attitudes toward Oriental White Stork-friendly farming, compared to 57.50% of those without such experience. Similarly, respondents with higher annual household income (71.13%) were considerably more optimistic than those with lower income (52.56%) (Figure 3). Taken together, these patterns indicate that both “capability-related factors” (having crossed technical/management barriers through prior adoption) and “resource-related factors” (higher income) are associated with higher acceptance, consistent with the idea that perceived feasibility and risk-bearing capacity condition willingness to participate.

We further assessed the effects of external economic incentives—price premiums and subsidy policies—on willingness to adopt Oriental White Stork-friendly farming. The findings indicated that about 50.45% of respondents were willing to adopt such practices if the purchase price was doubled compared to conventional rice. An additional 47.73% would be willing to adopt if the government compensated 70% of yield losses. Nevertheless, a significant proportion of respondents remained resistant: 35.45% reported they would not adopt regardless of government compensation, and 38.18% would remain unwilling even with substantial increases in market price. This distribution suggests that willingness is not uniformly “incentive-responsive”: while sizable shares respond to either market-based premiums or public compensation, a persistent segment remains unwilling under both scenarios, implying that perceived operational risks and labor demands may dominate monetary considerations for these farmers.

### 3.2. Willingness of Urban Respondents to Accept Environmentally Friendly Rice Planting Models and Influencing Factors

**Figure 4:**
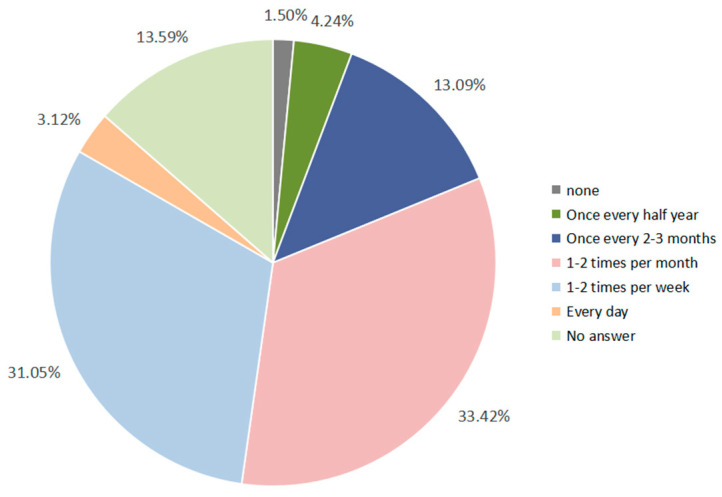
Pie chart of the frequency of respondents purchasing ecological agricultural products.

To assess urban respondents’ consumption habits regarding eco-labeled agricultural products, we asked a series of questions, including: “What proportion of ecological agricultural products currently available on the market do you believe are genuinely qualified?”; “How frequently do you purchase ecological agricultural products?”; and “What are your main reasons for purchasing such products?” The results suggested strong representativeness, as only 1.5% of respondents reported never purchasing eco-labeled products (Figure 4). However, 76.43% of respondents believed that nearly one-third of ecological agricultural products on the market were unqualified. Motivations for purchasing eco-labeled products were diverse: 34.47% associated the Oriental White Stork habitats with healthier and better-tasting food; 34.60% emphasized personal values, expressing a preference for supporting environmentally friendly and sustainable production; and 30.86% highlighted the potential role of such products in conserving endangered bird species. Overall, This results indicate a clear consumer base for eco-labeled products alongside pronounced concerns about label credibility, implying that demand and trust do not necessarily co-occur.

**Figure 5:**
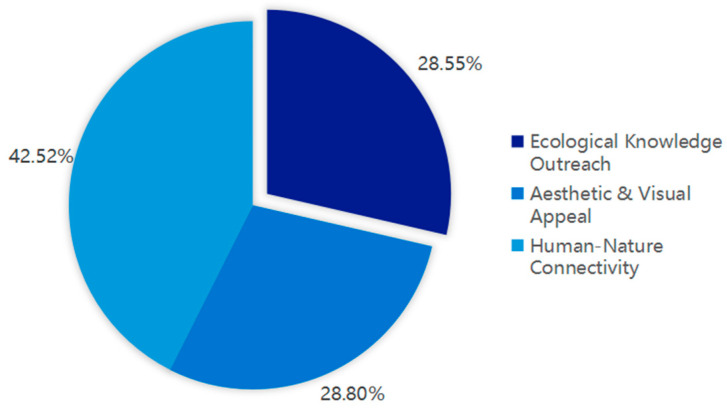
Public perceptions on the most effective strategies for raising awareness of environmental indicator species. Note: Percentages represent the proportion of respondents who selected each method. “Human-Nature Connectivity” refers to emphasizing the link between species and human living environments; “Aesthetic & Visual Appeal” involves enhancing species’ attractiveness through design; "Ecological Knowledge Outreach" focuses on increasing scientific information in publicity; In addition to the aforementioned options, others (0.12%) is omitted from Figure due to its small share.

To enhance the market appeal of ecological agricultural products and highlight their conservation value, this study explored consumer preferences for endorsement species in wildlife-friendly labeling [36]. Respondents were asked: “If a wildlife-friendly agricultural product were promoted using both a charismatic flagship species (e.g., the crested ibis) and an ecological indicator species (e.g., Anura), which would you be more inclined to purchase?” The results showed that a majority preferred products featuring indicator species (38.28%). Furthermore, 42.52% of respondents indicated that emphasizing the close link between these species and human living environments could enhance public awareness of the ecological importance of indicator species (Figure 5). Figure 5 therefore suggests that, for a notable share of consumers, perceived ecological “signal value” (i.e., species implying broader ecosystem condition) may be at least as salient as charisma-based appeal, which is relevant for designing conservation-oriented labeling strategies.

### 3.3. Preferences of Rural and Urban Respondents for Oriental White Stork-Friendly Rice Planting Models

#### 3.3.1. Rural Areas

The MNL results (Table 2) showed that farmers had significant preferences (*p* < 0.01) for the attributes rice planting models, rice-fish co-cropping system, rice palatability, and rice price. Except for price, all coefficients were negative. This indicated a preference among rural respondents for maintaining current planting practices, a reluctance toward raising fish in rice fields, and a favor for rice of average palatability quality—even while showing a willingness to accept higher rice prices. Notably, the presence of the Oriental White Stork in rice paddies was not significant in the pooled MNL results, suggesting that, on average, farmers’ stated choices were more strongly structured by practice-change and product/price considerations than by stork presence per se.

Rural respondents’ preferences for Oriental White Stork-friendly rice planting models exhibited significant heterogeneity, with two distinct latent groups identified. The first group (LCM1), comprising 53.9% of the sample, preferred no presence of the Oriental White Stork in rice fields (*p* < 0.001), favored rice with average palatability (*p* < 0.001), and supported higher rice prices (*p* < 0.001). This group was predominantly younger males with lower educational attainment (*p* < 0.001). The behavior of LCM1 suggests a “pure economic incentive” orientation; they are willing to adopt green practices only if the financial return is guaranteed, while remaining hostile to the actual presence of wildlife.

The second group (LCM2), comprising 46.1% of respondents, exhibited distinctly different preferences: they strongly favored the presence of Oriental White Storks in rice paddies (*p* < 0.01), showed no significant preference regarding rice palatability (*p* > 0.05), preferred to maintain current planting practices without raising fish in rice paddies (*p* < 0.001), and also supported higher rice prices (*p* < 0.001). This may reflect differences among groups in risk perception and tolerance, which become key factors hindering their participation in wildlife-friendly agricultural practices. LCM2 thus appears comparatively wildlife-tolerant but still practice-conservative (particularly regarding rice–fish co-cropping), suggesting that acceptance of biodiversity outcomes does not necessarily translate into acceptance of all associated practice changes.

#### 3.3.2. Urban Areas

Overall, urban respondents demonstrated significant preferences (*p* < 0.001) for the attributes rice planting models, presence of the Oriental White Stork in rice paddies, rice palatability, and rice price (Table 3). Except for price, all other attribute coefficients were significantly positive (*p* < 0.001), indicating that urban respondents strongly favored rice cultivated using environmentally friendly practices (e.g., organic or green planting), preferred rice with better palatability, and supported the presence of the Oriental White Stork in rice fields, while preferring not to pay excessively high prices. This pattern indicates a “pro-environment” orientation among consumers coupled with clear price sensitivity, consistent with the notion that urban support is contingent on affordability.

Based on the LCM analysis, four groups of urban respondents with distinct preference patterns were identified. LCM2 (22.2%) and LCM3 (70%) were predominantly younger respondents and largely mirrored the overall trends. Compared with the aggregate results, LCM2 showed no significant preference for planting models (*p* > 0.05), while LCM3 showed no significant preference regarding the presence of the Oriental White Stork in rice fields. LCM1 (1.2%) and LCM4 (6.6%) were more distinctive. LCM1 demonstrated no significant preference for any of the five attributes (*p* > 0.05), suggesting indifference toward ecological agricultural products. By contrast, LCM4 displayed significant preferences for all attributes (*p* < 0.01), but only rice planting models and price were positive. This indicated that LCM4 respondents were willing to pay premium prices for rice cultivated using organic or green methods, yet strongly opposed both the presence of the Oriental White Stork in rice paddies (*p* < 0.01) and the practice of rice-fish co-cropping system (*p* < 0.01). The LCM4 group may have already developed an interest in ecological products and previously purchased such items, representing a segment with established consumption habits that is willing to make sustained financial commitments to support environmental outcomes. Importantly, the LCM results reveal that even among urban consumers who value eco-friendly planting, biodiversity-linked attributes (stork presence) can be divisive for a small but non-negligible segment (LCM4), underscoring that “eco-product preference” and “wildlife presence preference” are not always aligned.

## 4. Discussion

The rapid expansion of green and organic agriculture across the Sanjiang Plain offers new opportunities to protect endangered migratory species like the Oriental White Stork. Oriental White Stork-friendly farming, in particular, shows significant potential to ease tensions between conservation goals and economic development [3]. However, as local farmers gain more experience in sustainable practices and consumer demand for green products continues to rise, the preferences of both producers and consumers have begun to shift. Against this backdrop, a thorough understanding of the preferences of both urban consumers and rural residents in habitat areas toward environmentally friendly farming is crucial for designing effective conservation policies and sustainable agricultural strategies.

### 4.1. Preferences of Residents in the Oriental White Stork Habits of the Sanjiang Plain for Oriental White Stork-Friendly Rice Planting Models

Consistent with the findings of the 2018 study, nearly all respondents exhibited a positive preference for price [3]. The results indicate that a key factor reducing respondents’ willingness to participate is their past experience with environmentally friendly farming practices, such as green or organic methods, which have proven difficult to make profitable [37,38,39]. The main reasons include low profitability, high entry barriers, and substantial technical challenges [40], which are likely critical factors contributing to local communities’ reluctance to adopt such practices. However, the study suggests that the success of biodiversity management incentives, implemented through policy measures and regional strategies aimed at landscape-level integration, largely depends on a deeper understanding of farmers’ decision-making processes [23]. Only by enhancing ecological awareness, establishing long-term effective policy support, and improving market mechanisms can individual farmers be encouraged to participate.

Previous research has shown that rural respondents view environmentally friendly farming, such as green and organic farming, as significantly enhancing rice palatability, with tangible improvements in the final product. Concurrently, consumers have been found willing to buy a premium for such improvements [36]. However, as farmers gained more practical experience, their perception of this palatability benefit became more negative; this was particularly evident in groups such as LCM1. This shift occurred because their farming experience demonstrated that adopting these practices did not actually lead to perceptible improvement in rice palatability and the absence of such verifiable quality upgrades significantly reduced consumers’ willingness to purchase [41].

The accumulation of practical experience, particularly in rural areas, tends to heighten respondents’ perception of risk. Most farmers are unable to absorb potential losses from failed transitions. Coupled with recent declines in grain prices [42], environmentally friendly farming often reduces yields on leased land, further increasing economic risk. In the absence of policy or institutional support [43], such pressures may lead farmers to adopt a cautious or wait-and-see approach toward Oriental White Stork-friendly farming. Beyond economic risk, sensitivity to wildlife-related risks may also increase [44]. Farmers’ decision-making is a complex and often non-linear process, influenced by numerous external factors—social, landscape, community, and farm-level—as well as internal factors [23]. Mobilization and practice are likely to be more effective once risks are reduced to an acceptable level.

Groups with prior experience in environmentally friendly farming, who are relatively young and have higher household incomes, may be ideal candidates for piloting Oriental White Stork-friendly farming [45]. Such groups incur lower technical learning costs. However, our findings indicate that existing environmentally friendly farming in the region—such as rice-duck and rice-loach co-cropping systems—can conflict with current local certification standards, creating barriers to participation [46]. For instance, although the LCM2 group is willing to engage in environmentally friendly practices, they do not favor rice–fish co-cropping system. While this method allows limited use of low-toxicity pesticides and approved fertilizers, it often fails to meet strict organic standards, preventing fields from passing environmental inspections. Furthermore, although some respondents are willing to dedicate portions of their farmland to environmentally friendly farming, fragmented plots risk cross-contamination, whereas isolated plots may yield insufficient production and limited economic returns. According to Xu et al., this group may participate primarily out of public-mindedness or a risk-tolerant spirit [24], and they are likely to have a higher capacity to bear economic risks rather than expecting environmentally friendly farming to be profitable. In fact, the motivations of this group are similar to the reasons urban consumers purchase environmentally friendly agricultural products, leaning more toward being the compensator rather than the compensated party. Further joint analysis integrating this group’s income structure and underlying value orientations could provide deeper insights.

### 4.2. Preferences of Potential Urban Consumers for Oriental White Stork-Friendly Rice Planting Models

Consumers who have previously purchased environmentally friendly products remain willing to pay for agricultural products labeled as Oriental White Stork-friendly. In line with prior research [3,46], the findings show that consumers generally prioritize product quality and prefer rice that is both affordable and of superior palatability. However, with rising income levels and heightened environmental awareness among urban residents, some shifts in preferences have become evident. Notably, consumers in the LCM4 group display an obvious willingness to pay higher prices for organic and green products. Further evidence suggests that these consumers have already developed an interest in ecological products, possess established consumption habits, and are willing to make sustained financial commitments to support environmental protection.

The results suggest that the effectiveness of indicator species as product endorsers relies on consumers having a clear understanding of the species. Consistent with previous studies, biological indicators are perceived as offering more direct and tangible evidence of environmental protection outcomes [47]. Consumers tend to place greater trust in products endorsed by environmental indicator species that are closely connected to human living environments, such as Anura and Lampyridae [48]. Nevertheless, although respondents indicated a willingness to pay a premium for products labeled with such species, they maintained a negative attitude toward the presence of Oriental White Storks in rice fields. This indicates a potential misunderstanding of the Oriental White Stork’s ecological role, with some consumers even perceiving it as a possible risk to water quality or product safety. Such misperceptions may stem from a contrast with wildlife-friendly certifications endorsed by highly recognizable “charismatic species” (e.g., the giant panda), whose ecological and symbolic value are well established in the public consciousness. By contrast, the Oriental White Stork, while serving as an indicator of wetland ecosystem health, has not yet been widely recognized by the public for its ecological functions and conservation importance.

### 4.3. Challenges in Implementing Oriental White Stork-Friendly Farming in the Sanjiang Plain

In recent years, the area dedicated to organic and green farming in the Sanjiang Plain has steadily expanded, accompanied by a concurrent increase in the Oriental White Stork population [49]. This expansion, however, has also raised the conservation costs borne by farmers engaged in traditional intensive agriculture. At the same time, growing consumer awareness and willingness to pay for environmentally friendly agricultural products present new opportunities. Specifically, Oriental White Stork-friendly farming provides a potential means of reconciling the dual objectives of the Oriental White Stork conservation and organic farming, while alleviating inequities in the distribution of conservation burdens. Leveraging the existing foundation of environmentally friendly agricultural practices could greatly enhance the efficiency of this transition.

The underdeveloped markets for ecological products and inconsistent quality standards remain major obstacles to stakeholder participation. Evidence from research and practice underscores [50] that a unified certification system and standardized implementation procedures are critical to ensuring engagement, whereas vague or overly stringent requirements deter willing stakeholders [48]. Securing government or NGO investment, establishing risk-sharing mechanisms, and mitigating production risks could encourage farmers’ involvement while strengthening consumer confidence [51]. Furthermore, considering the disadvantaged socioeconomic conditions of local respondents, access to professional technical guidance would substantially enhance the feasibility of implementing Oriental White Stork-friendly farming.

A lack of consumer awareness regarding wildlife-friendly farming remains a major barrier to its feasibility. At present, many consumers view wildlife conservation and agricultural production as entirely separate domains [3,51]. Even when some are willing to pay for environmental outcomes, the limited availability of information makes it difficult for them to recognize the connection [52]. Systematic educational efforts that correct these misconceptions and promote the concept of “conservation-production synergy” are therefore critical for increasing social acceptance of Oriental White Stork-friendly farming. In the early stages of implementation—when awareness is generally low—emphasis should be placed on widely recognized environmental benefits, such as biodiversity conservation and ecosystem health. Highlighting well-known environmental indicator species can help consumers perceive the link between effective wildlife protection and improved ecological outcomes. Complementary environmental monitoring systems can further build consumer trust, enhancing the feasibility of Oriental White Stork-friendly agricultural practices.

Social identity has a profound influence on cognition, as individuals interpret reality through the lens of their socially defined self-concepts [22]. Diversified policy approaches are therefore more likely to be feasible and effective [53]. Even among groups with similar social backgrounds, preferred solutions may differ, often for reasons that are not entirely rational, as highlighted by Simpfendorfer et al. [54]. Addressing these complex factors requires moving beyond purely socio-economic characteristics, identifying distinct stakeholder profiles, and designing policy packages that are as harmonious and integrative as possible. To date, most research has focused on one-off experiments across different groups, overlooking the lack of long-term repeated surveys or respondent tracking [55] and the establishment of long-lasting policies. In reality, both urban and rural residents rely heavily on long-standing community culture and traditional norms [22,23,56]. The success of biodiversity management incentives implemented through policy and regional strategies for landscape integration largely depends on a deeper understanding of farmers’ decision-making processes [23]. Research also indicates that urban residents rely more on government policies and green infrastructure, whereas rural residents’ green perceptions are primarily shaped by community atmosphere and direct environmental experiences [57]. Focusing solely on short-term economic incentives may expose stakeholders to multiple uncertain economic risks, thereby hindering their sustained participation. Overall, the significant differences in preferences for Oriental White Stork-friendly rice planting models between urban and rural respondents reveal potential policy complementarities. Promoting such practices requires coordinated efforts targeting both consumers and producers, along with the establishment of tailored supply chains that align with their respective demands and incentives. For instance, while the rural LCM1 group opposed the presence of the Oriental White Stork in rice paddies, their primary concern was obtaining higher rice prices, showing reserved attitudes toward environmentally friendly farming methods. In contrast, the urban LCM4 group strongly preferred organic and green production modes and was willing to pay a premium yet objected to the presence of the Oriental White Storks. The rural LCM1’s desire for higher prices could serve as an economic incentive to adopt green and organic practices, thereby helping to meet the urban LCM4’s high demand for such products. Similarly, the rural LCM2 group showed positive attitudes toward the presence of the Oriental White Stork while preferring to maintain traditional farming methods, reflecting the urban LCM2 group’s preference for rice fields hosting Oriental White Storks without a significant preference for environmentally friendly farming. This complementary relationship calls for the development of an Oriental White Stork-centered value chain that integrates these divergent preferences. Such a market-based approach, supported by well-targeted policies, could provide a solid foundation for differentiated development of Oriental White Stork-friendly farming, achieving a win-win outcome that increases farmer income and conserves ecosystems.

## 5. Conclusions

This study applies a Choice Experiment to examine preference heterogeneity toward Oriental White Stork-friendly farming in the Sanjiang Plain, filling a gap in the literature on long-term and multi-group analyses under changing policy and experiential contexts. The results reveal clear cognitive and behavioral differences between urban consumers and rural residents: while some urban consumers are willing to pay a premium for ecological products, misconceptions about the presence of storks persist, whereas rural groups respond differently to price incentives and intrinsic conservation motivations. These findings indicate that Wildlife-Friendly Farming cannot be effectively promoted through a one-size-fits-all approach. Instead, stratified, group-specific policy and market mechanisms that align producer incentives with consumer demands are essential to support the long-term viability of biodiversity-friendly agricultural systems.

## Figures and Tables

**Table 1:** Attributes and their levels in the choice experiment.

Attributes	Levels	Description
Rice Planting models	Conventional	The rice cultivation model directly influences growers’ agricultural practices and economic outcomes while determining the ecological impact of farming activities. For consumers, environmentally friendly farmland is typically perceived as being synonymous with high environmental standards and enhanced food safety, which significantly shapes purchasing decisions. Drawing on China’s rapidly expanding green and organic cultivation paradigms, this study incorporates three levels to reflect stakeholder acceptability and trust across a gradient of ecological certification requirements.
	Organic	
	Green/pollution-free	
Rice-fish co-cropping system	No	By simulating natural wetland functions, rice–fish co-culture systems provide critical foraging habitats for the Oriental Stork. Consumers typically perceive such systems as indicators of superior water quality and higher rice quality. In this study, a binary level (“Yes” vs. “No”) was designed to explore respondents’ preferences for the rice–fish co-culture system while minimizing their cognitive load.
	Yes	
Presence of the Oriental White Stork in rice paddies	No	As a regional indicator species, the presence of the Oriental Stork in agricultural fields serves as a direct reflection of the efficacy of “wildlife-friendly” farming practices. Furthermore, this attribute functions as a proxy to measure the cognitive conflict between conservation costs (e.g., economic losses resulting from bird damage) and perceived conservation value. For this attribute, a binary level (“Yes” vs. “No”) was established within the experimental design.
	Yes	
Rice palatability	Average	Palatability is a pivotal commercial attribute that bridges production-side returns with consumer demand, directly determining the market competitiveness and long-term brand viability of rice products. To minimize the cognitive load of respondents, this study established a binary level (“Average” vs. “Good”) for this attribute.
	Good	
Suggested retail price for Oriental White Stork-friendly rice	6 ¥/kg	Price is a critical determinant of both farmers’ motivation to participate and consumers’ purchasing decisions. Based on the current market prices for organic rice in China, this study incorporates four distinct price levels.
	16 ¥/kg	
	26 ¥/kg	
	36 ¥/kg	

**Table 2:** Preference of farmers around the Oriental White Stork habitat for the Oriental White Stork-friendly rice planting models.

Attribute Levels	MNL	LCM1 (53.9%)	LCM2 (46.1%)
ASC	−1.09754 *** (0.15285)	−13.5610 (28.71777)	−1.34820 *** (0.20352)
Rice planting models	−0.09817 ** (0.04355)	0.09575 (0.12225)	−0.92270 *** (0.11849)
Rice-fish co-cropping system	−0.57652 *** (0.08995)	−0.01968 (0.25358)	−2.69327 *** (0.21326)
Presence of the Oriental White Stork in rice paddies	−0.08939 (0.10000)	−1.29962 *** (0.31692)	0.49442 ** (0.19460)
Rice palatability	−0.13671 *** (0.05064)	−1.37247 *** (0.34338)	0.00047 (0.06076)
Willingness to Pay for rice	0.12830 *** (0.00998)	0.38652 *** (0.04158)	0.10215 *** (0.01840)
Gender		1.06592 *** (0.27098)	
Age		−0.66185 ** (0.25569)	
Education		−0.64818 ** (0.27424)	

Note: Confidence intervals are 95%. Significance levels are indicated as: * *p* < 0.05, ** *p* < 0.01, *** *p* < 0.001.

**Table 3:** Consumers’ preference for Oriental White Stork-friendly rice planting models in urban areas.

Attribute Levels	MNL	LCM1 (1.2%)	LCM2 (22.2%)	LCM3 (70.0%)	LCM4 (6.6%)
ASC	−1.19125 *** (0.05417)	−8.45768 (35.79827)	−0.09577 (0.20606)	−2.74801 *** (0.23258)	0.96850 *** (0.24449)
Rice planting models	0.25512 *** (0.03271)	−8.61776 (3098.461)	0.06823 (0.11038)	0.26031 *** (0.04920)	6.86375 *** (2.29431)
Rice-fish co-cropping system	−0.06023 (0.08114)	17.0278 (3097.613)	−0.19904 (0.21248)	−0.08152 (0.13022)	−5.10696 ** (2.11822)
Presence of the Oriental White Stork in rice paddies	0.31573 *** (0.09554)	41.3897 (6230.975)	0.70924 *** (0.23497)	0.16721 (0.15835)	−13.6041 ** (5.29662)
rice palatability	0.54695 *** (0.06519)	24.6573 (6195.653)	0.68202 *** (0.18162)	0.52421 *** (0.10991)	−8.42380 *** (3.19469)
Willingness to Pay for rice	−0.04520 *** (0.00867)	−0.21313 (0.41951)	−0.07158 *** (0.02111)	−0.04205 *** (0.01301)	0.38150 ** (0.14884)
Gender		1.59400 (2.07557)	0.49774 (0.56759)	0.15281 (0.46298)	
Age		−1.65095 (2.35098)	−0.87072 ** (0.36028)	−0.69104 *** (0.26436)	
Education		2.96614 (4.94775)	0.28497 (0.40925)	0.20352 (0.30495)	

Note: Confidence intervals are 95%. Significance levels are indicated as: * *p* < 0.05, ** *p* < 0.01, *** *p* < 0.001.

## Data Availability

The data presented in this study are available on request from the author.
